# 762 Improving Pain Management During Burn Wound Care Procedures

**DOI:** 10.1093/jbcr/irae036.304

**Published:** 2024-04-17

**Authors:** Jason Sheaffer, Monica Hutson, Steven E Wolf, Carlos Jimenez, Sharron Forest

**Affiliations:** University of Texas Medical Branch, Blocker Burn Unit, Galveston, TX; University of Texas Medical Branch, Galveston, TX; University of Texas Medical Branch, Blocker Burn Unit, Galveston, TX; University of Texas Medical Branch, Galveston, TX; University of Texas Medical Branch, Blocker Burn Unit, Galveston, TX; University of Texas Medical Branch, Galveston, TX; University of Texas Medical Branch, Blocker Burn Unit, Galveston, TX; University of Texas Medical Branch, Galveston, TX; University of Texas Medical Branch, Blocker Burn Unit, Galveston, TX; University of Texas Medical Branch, Galveston, TX

## Abstract

**Introduction:**

During burn wound care, the intention is to manage pain throughout the procedure with minimal to no sedation. While it is rare that burn patients experience zero pain, 25% of patients were experiencing excessive pain with self-reported pain scores of ≥7 throughout their procedure. Identified gaps included nursing documentation of intra-procedure pain scores and provider variability on pain management.

**Methods:**

A multidisciplinary Pain Management Task Force developed a Pain Management Guideline (PMG) comprised of frequent pain assessment, nonpharmacologic techniques, and pharmacologic therapy of nonopioids, opioids, and sedation. The established PMG consisted of a pain assessment at the start of the procedure, repeating every 15 minutes until procedure completion. Music therapy provided distraction at the patient’s discretion. Nitrous oxide utilization provided a nonopioid intervention. Building an order set allowed PMG incorporation into the electronic health record (EHR).

**Results:**

Within 20 weeks, the percentage of patients with a mean procedural pain score of ≥7 reduced from 25% to 21%, short of the intended goal of 15%. The predominant approach to pain management remains the use of narcotic opioids. There was low usage of nitrous oxide with the unanticipated high prevalence of exclusionary factors of NPO compliance, facial trauma, and impairment. However, nitrous oxide was effective 90% of the time in reducing pain scores to < 7. Moderate sedation procedures proved a challenge in case coordination while maintaining regulatory compliance. Nursing documentation of pain scores improved by 79% after modification of the standard nursing note to include an intra-procedure pain assessment, which also increased the average number of pain assessments from 2 to 3. Encouragingly, PMG implementation did not increase the average procedure time or patient safety events.

An unanticipated finding was the presence and impact of Social Determinants of Health (SDOH) on pain perception and management. Of the patients reporting excessive pain, several had a pre-procedural pain score ≥7, which remained elevated throughout the intra- and post-procedure intervals. Positive SDOH factors identified included a significant history of substance abuse, homelessness, poverty, and mental health disease.

**Conclusions:**

Pain management principles were codified and organized into a PMG. Development and implementation of a PMG decreased self-reported pain scores for burn patients. The guideline improved pain management consistency between providers and improved nurses’ assessment and documentation of pain scores. When considering the practical implications, it is essential to understand how SDOH may impact pain management throughout all stages of care for burn patients.

**Applicability of Research to Practice:**

A quality improvement project to reduce the rate of mean self-reported procedural pain scores of ≥7 during burn wound care procedures.

